# Risk-Taking Behaviors in Iranian Children and Adolescents: A Latent Class Analysis Approach: Caspian IV Study

**Published:** 2018-09-23

**Authors:** Abbas Abbasi-Ghahramanloo, Ramin Heshmat, Saeid Safiri, Mohammad Esmaeil Motlagh, Gelayol Ardalan, Armita Mahdavi-Gorabi, Hamid Asayesh, Mostafa Qorbani, Roya Kelishadi

**Affiliations:** ^1^ Health Management Research Center, Baqiyatallah University of Medical Sciences, Tehran, Iran; ^2^ Department of Epidemiology, School of Public Health, Iran University of Medical Sciences, Tehran, Iran; ^3^ Chronic Diseases Research Center, Endocrinology and Metabolism Population Sciences Institute, Tehran University of Medical Sciences, Tehran, Iran; ^4^ Department of Public Health, School of Nursing and Midwifery, Maragheh University of Medical Sciences, Maragheh, Iran; ^5^ Department of Pediatrics, Ahvaz Jundishapur University of Medical Sciences, Ahvaz, Iran; ^6^ Department of School Health, Bureau of Population, Family and School Health, Ministry of Health and Medical Education, Tehran, Iran; ^7^ Department of Basic and Clinical Research, Tehran Heart Center, Tehran University of Medical Sciences, Tehran, Iran; ^8^ Department of Medical Emergencies, Qom University of Medical Sciences, Qom, Iran; ^9^ Non-Communicable Diseases Research Center, Alborz University of Medical Sciences, Karaj, Iran; ^10^ Endocrinology and Metabolism Research Center, Endocrinology and Metabolism Clinical Sciences Institute, Tehran University of Medical Sciences, Tehran, Iran; ^11^ Child Growth and Development Research Center, Research Institute for Primordial Prevention of Non-communicable Disease, Isfahan University of Medical Sciences, Isfahan, Iran

**Keywords:** Risk behaviors, Children and adolescents, Students, Iran

## Abstract

**Background:** Risk taking behaviors have several negative consequences. This study aimed to identify the subgroups of students based on risk-taking behaviors and to assess the role of demographic characteristics, depression, anxiety, socioeconomic status (SES), physical inactivity and screen time on membership of specific subgroup.

**Study design:** Cross-sectional study.

**Methods:** This nationwide survey was conducted in 2011-2012 among 14880 students, aged 6-18 yr, selected by multistage, cluster-sampling method from 30 provinces of Iran. The students completed two sets of anonymous and validated questionnaires, obtained from the World Health Organization-Global School Health Survey questionnaires. Latent class analysis was performed to achieve the study objectives.

**Results:** Overall, 13486 children and adolescents participated were enrolled (response rate 90.6%). They consisted of 50.8% boys, with a mean age of 12.47 ±3.36 year. The prevalence of physical fight, bullying, victimization, active smoking, passive hookah and passive cigarette smoking was 39.7%, 17.4%, 27.2%, 5.9%, 21.1 and 33.8%, respectively. Five latent classes were identified: (a) low risk (46.7%), (b) passive smoker (25.2%), (c violence and aggression taker with passive smoking (13.5%), (d) violence and aggression taker without passive smoking (10.8%) and (e) high risk (3.8%). Higher age (OR=1.41), being male (OR=5.21), depression (OR=4.58), anxiety (OR=3.38) and screen time (OR=3.11) were associated with high-risk class.

**Conclusion:** The prevalence of some risk-taking behaviors among Iranian students is high. Our findings emphasize the importance of planning and evaluating preventive interventions by considering different high-risk behaviors simultaneously

## Introduction

In spite of promotion of health efforts, adolescents and young adults continue to engage in risk-taking behaviors^1^. These behaviors have direct links to chronic disease. Enhanced health and lessen the risk of chronic illnesses can be resulted through the modification of these risk-taking behaviors^1,2^.

A great deal of risk-taking researches focused on the negative consequences of risk-taking behaviors in adolescent. For example smoking, bullying, physical fight, reckless behavior, criminal activities, heavy drinking, drug use, and reckless driving are regarded as potentially risky behaviors that may have negative long-term consequences^3-6^. Regarding these negative consequences of risk-taking behavior has become one of the most effective approaches in the adolescent's studies^7^.

Identifying subgroups of adolescents based on risk-taking behaviors allows health service providers and policy makers to recognize individuals who have identical characteristics based on patterns of risky behaviors^8^.

For such distinctions between individuals, a person-centered analytic approach is a useful and interesting statistical method ^9^. In identifying distinct sub-groups, latent class analysis (LCA) uses categorical and cross-sectional observed indicators to assign class memberships to individuals and yields unobserved (latent) classes of people in an attempt to arrive at the smallest number of latent classes^7-9^.

Understanding the pattern of risky behaviors that adolescents engaged in is necessary for consideration intervention strategies. Adolescents who engage only in drug use, for instance, may be somewhat different from those involved with a spectrum of risky behaviors^10^.

A few studies have used latent classes to represent subgroups of adolescent risk behaviors. Latent class analysis was used to identify adolescent risk behavior subgroups^11^. The authors classified students into four classes, comprised of varying types and degrees of risky behavior. Specifically, there were two groups that ‘‘abstained’’ and ‘‘experimented’’ with risky behaviors and two others that had higher patterns of such activities.

In this study we used a latent class analysis to identify subgroups of students. These subgroups are made based on the student’s response to each question about risk taking behaviors. The present study used a latent class analysis to investigate potential subgroups of students based on their responses to a series of questions about risky behaviors. Therefore, the aim of this study was to (a) identify the subgroups of students on the basis of risk-taking behaviors, (b) document the prevalence of the subgroups, and (c) assess the role that demographic characteristics, depression, anxiety, socioeconomic status (SES), physical inactivity and screen time may play in forming the classifications.

## Methods

### Study population and sampling framework

This study was as the fourth survey of a national school-based surveillance program, entitled “Childhood and Adolescence Surveillance and Prevention of Adult Non-communicable Disease (CASPIAN-IV)” study conducted in 2011-2012 among 14880, aged 6-18 yr students in urban and rural areas of 30 provinces in Iran. The students were selected by multistage, cluster sampling method from provinces of Iran (48 clusters of 10 students in each province). Stratification was performed in each province according to the residence area (urban/rural) and school grade (elementary/intermediate/high school). The sampling was proportional to size with equal sex ratio; i.e., equal number of boys and girls were selected from each province and the ratios in urban and rural areas were proportionate to the population of urban and rural students. In this way, the number of samples in rural/urban areas and in each school grade was divided proportionally to the population of students in each grade^12^.

Cluster sampling with equal clusters was used in each province to reach the necessary sample size. Clusters were determined at the level of schools, including 10 sample units (students and their parents) in each cluster^12^.

The sample size was determined according to the cluster sampling method and to achieve a good estimate of the main risk factors of interest such as physical inactivity. The maximum sample size that could give a good estimate of all risk factors of interest was selected. Thus, the sample size was calculated as 480 subjects in each province. Overall, 48 clusters of 10 subjects in each of the provinces and 14880 students and an equal number of their parents were selected from 30 provinces^12^.

### Study tools

We completed two sets of questionnaires for students and the parents. These questionnaires were obtained from Global School Health Survey (GSHS) and then were converted into Persian. The reliability and validity of them were previously assessed^13^.

Six dichotomous variables were used to assess risk-taking behaviors. The variables were (a) physical fight (History of ≥1 times fight during the last 12 months), (b) bullying (History of ≥1 times bullying during the last 3 months), (c) victimization (History of ≥1 times fight during the last 3 months in the school), (d) active smoking (currently use), (e) passive hookah smoking, and (f) passive cigarette smoking.

The method and variables used for calculating of SES were approved in the International Reading Literacy Study (PIRLS)^14^. SES of family was calculated according to Principal Component Analysis (PCA) method by using some variables including parent education and job, type of school (private or governmental) and family assets (private car and computer). Students were classified into low, moderate and high SES based SES score.

### Ethical considerations

The Ethics Committee of Tehran University of Medical Sciences and Isfahan University of Medical Sciences approved the study protocol and its questionnaire. To enhance the validity of student’s self-reports, an explanation was presented by interviewers about the aims of the study, anonymity of the questionnaires, and the voluntary nature of participation in the survey. Furthermore written informed consent and verbal consent was obtained from the parents and students, respectively.

### Statistical analysis

The LCA method was used in data analysis. First, the authors used descriptive statistics to examine characteristics of indicators that used to determine the number of latent classes. In the second step, we tested the LCA models with classes ranging from 1 to 8 using PROCLCA statement. In order to investigate model identification, each latent class model was fit to the data 50 times by different random starting values. By various iterations for the number of identified classes of the latent variable and comparing the frequencies of the observed response patterns with the expected ones, the LCA determines the best model and calculates a statistics similar to χ^2^ called G2. Based on G2 statistic, Akaike Information Criterion (AIC) and Bayesian Information Criterion (BIC) can be calculated for model selection. For all information criteria, a smaller value represents a more optimal balance of model fit and parsimony; thus, a model with the minimum AIC or BIC might be selected. When the *P* value is significant, we cannot select this model, because the observed and expected response patterns differ from each other significantly. Thus we could not select 1-4 latent class model. Between other models, 5 class model had lowest AIC and BIC. Actually, we selected this model based on the AIC and BIC.

In the final step, after finalizing model, we entered gender, resident place, depression (being permanently sad for two weeks in a way that prevents the daily activities during the last 12 months), anxiety (being anxious in the past six month), socioeconomic status (weak), physical inactivity (at least 30 min physical activity in none of days of week) and screen time (above 2 h television watching and working with computer) as covariates in the LCA model by using multinominal regression. Analyses were conducted using Proc LCA in SAS 9.2 software (SAS Institute Inc. Cary, NC, USA).

## Results

The study participants were 13486 students out of 14880 invited subjects (participation rate of 90.6%). They consisted of 49.24% girls, 75.6% urban residents, with a mean age of 12.5 yr (SD: 3.36). A summary of risk-taking behaviors is shown in Table 1. The prevalence of some of the risk-taking behaviors (e.g., physical fight) was higher than the others.

**Table 1:** Percentages of students responding “Yes” to questions about risk-taking behaviors

**Items**	**Number**	**Percentage (95% CI)**
Physical fight	5352	39.7 (38.9, 40.5)
Bullying	2347	17.4 (16.8, 18.0)
Victimization	3670	27.2 (26.5, 27.9)
Active smoking	790	5.9 (5.5, 6.2)
Passive hookah smoking	2850	21.1 (20.4, 21.8)
Passive cigarette smoking	4557	33.8 (32.9, 34.6)

With considering six dichotomous indicators, there were 64 possible response patterns. We attempted to fit the LCA models with classes ranging from 1 to 8. For each LCA model, G2, AIC, and BIC are shown in Table 2.

**Table 2:** Comparison of latent class analysis models with different latent classes based on Model Selection Statistics

**No. of** **latent class**	**No. of parameters** **estimated**	**G** ^ 2 ^	**df**	**AIC**	**BIC**	***P*** **value**
1	6	4660.94	57	4672.94	4718.00	0.001
2	13	1062.12	50	1088.12	1185.74	0.001
3	20	322.48	43	362.48	512.67	0.001
4	27	94.76	36	151.76	354.52	0.001
5	34	31.49	29	99.49	354.81	0.343
6	41	18.19	22	100.19	408.08	0.695
7	48	10.95	15	106.95	467.40	0.756
8	55	7.75	8	117.75	530.77	0.458

AIC: Akaike information criterion; BIC: Bayesian information criterion

According to these model selection criteria, the five latent classes’ model was appropriate. The results of the five LCA classes model showed that differences between the expected and observed frequency of response patterns were not statistically significant (G2=31.49, df=29, *P*-value=0.34). The results of five latent classes’ model are shown in Table 3, which includes latent class prevalence and item-response probabilities. Table 4 shows the predictors (covariates) of membership in latent classes of risk-taking behaviors by considering the low-risk class as a reference group.

**Table 3:** The five latent class model of risk-taking behaviors

**Latent class**	**Low-risk**	**Passive smokers**	**Violence and aggression taker**		**High-risk**
			**Passive smokers**	**Not passive smokers**	
	**Rho estimate (SE)**	**Rho estimate (SE)**	**Rho estimate (SE)**	**Rho estimate (SE)**	**Rho estimate (SE)**
**Latent class prevalence**	0.467 (0.044)	0.252 (0.044)	0.135 (0.023)	0.108 (0.025)	0.038 (0.013)
**Item-response probabilities (Yes)**					
Physical fight	0.201 (0.012)	0.382 (0.022)	0.726 (0.022)	0.691 (0.026)	0.794 (0.029)
Bullying	0.014 (0.006)	0.037 (0.013)	0.571 (0.029)	0.566 (0.033)	0.544 (0.039)
Victimization	0.105 (0.007)	0.121 (0.011)	0.784 (0.041)	0.641 (0.035)	0.468 (0.034)
Active Smoking	0.014 (0.003)	0.066 (0.011)	0.000 (0.003)	0.052 (0.013)	0.783 (0.266)
Passive hookah smoking	0.029 (0.020)	0.469 (0.052)	0.376 (0.027)	0.042 (0.076)	0.637 (0.037)
Passive cigarette smoking	0.171 (0.023)	0.585 (0.042)	0.640 (0.097)	0.010 (0.040)	0.608 (0.038)

***Note***. The probability of a “No” response can be calculated by subtracting the item-response probabilities shown above from 1.

The probability of membership in each latent class is shown in the first row of Table 3. Nearly 47% and 4% of students were low risks and high risk, respectively.

The conditional probabilities of a “Yes” response to each risk-taking behavior are listed in Table 3. These probabilities form the basis for interpretation and labeling of the latent classes. The larger conditional probabilities are in bold to highlight the overall pattern. Latent Class 5, high risk, was characterized by a high probability of responding “Yes” to all of the risk-taking behaviors. The probability of victimization in this class is very near to the cut off value (0.5).

Individuals in this latent class were likely to report that they had engaged in all risk-taking behaviors. In contrast, those in Latent Class 1, low risk, were likely to report not having engaged in any of the risk-taking behaviors. Three other latent classes reflect different patterns of risk-taking behaviors. Latent Class 2, passive smoker, had a relatively high probability of reporting passive cigarette smoking. The probability of passive hookah smoking is near to the cut in value. Latent Class 3, violence and aggression taker with passive smoking had a high probability of reporting four type of risk-taking behavior, namely, physical fight, bullying, victimization, and passive smoking. Latent class 4, violence and aggression taker without passive cigarette and hookah smoking, had a high probability of reporting three behaviors.

The adjusted odds ratio indices of membership in each class, compared to the first class, and associated with the independent variables are also listed in Table 4. For example, being male, compared to being female, increases the odds of membership in Classes 2, 3, 4 and 5, compared to Class 1. Similarly having depression and anxiety notably increases the odds of membership in all latent classes compared to Class 1. The value of ORs, as well as 95% confidence interval for other covariates, is shown in Table 4.

**Table 4:** Predictors of membership in latent classes of risk taking behaviors

**Predictors**	***P*** ** value**	**Passive smokers**	**Violence and aggression taker**		**High risk**
			**Passive smokers**	**Not passive smokers**	
		**OR (95% CI)**	**OR (95% CI)**	**OR (95% CI)**	**OR (95% CI)**
Age (year higher)	0.001	1.01 (0.97, 1.04)	1.02 (0.98, 1.04)	0.95 (0.92, 0.98)	1.41 (1.33, 1.49)
Gender (being male)	0.001	1.96 (1.53, 2.52)	3.45 (2.74, 4.33)	3.11 (2.49, 3.88)	5.21 (3.91, 6.94)
Being urban citizen	0.325	0.91 (0.75, 1.10)	0.91 (0.72, 1.15)	0.83 (0.69, 0.98)	0.98 (0.72, 1.33)
Depression	0.001	2.58 (1.85, 3.59)	2.66 (1.89, 3.73)	4.49 (3.34, 6.04)	4.58 (3.23, 6.51)
Anxiety	0.001	2.17 (1.68, 2.79)	2.17 (1.64, 2.86)	3.34 (2.63, 4.23)	3.36 (2.48, 4.53)
SES status (weak)	0.001	1.39 (1.16, 1.70)	0.88 (0.70, 1.10)	1.19 (1.00, 1.41)	0.85 (0.66, 1.12)
Physical inactivity	0.124	1.11 (0.83, 1.46)	0.87 (0.60, 1.24)	0.95 (0.72, 1.24)	0.67 (0.46, 0.99)
Screen time (high)	0.001	1.64 (1.23, 2.18)	2.00 (1.49, 2.70)	2.34 (1.82, 3.00)	3.11 (2.31, 4.19)

***Note.*** The reference class = latent class 1

## Discussion

The results of the study indicated the prevalence of each risk-taking behavior, namely, physical fight, bullying, victimization, active smoking, passive hookah, and cigarette smoking. Physical fight was common behavior with a prevalence of 39.7%.

The prevalence of three behaviors related to violence and aggression (physical fight, bullying, and victimization) was high in this study. These factors tend to be co-occurring with together. Depression and anxiety are highly associated with these behaviors (Tables 3,4).

Aggression is common among Iranian students and their teachers. In Iran, some students encounter with physical punishment in the school. These students tend to relate their negative behaviors such as aggression to the school infrastructure, horrible and unpleasant communication between scholl authorities and students and workload of school homework. In some schools, students encounter with physical punishment and tend to relate their aggressive behaviors to the school infrastructure, harsh and unpleasant communication style of school authorities with students and workload of school homework. On the other hand, teachers hardly accept their faulty interactions and tent to blame the external factors out of the school for their verbal aggression^15^.

Lack education and knowledge of Iranian families about the children’s emotional needs and mistakes in educational methods and faulty behaviors in schools could be related with high prevalence of violence-related behaviors in this study. It needs for more studies to assess the related factors to these phenomena.

The prevalence of childhood and adolescent depression in Iran was 43.55% using the Beck Depression Inventory (BDI), 15.87 % using Symptoms Checklist-90 (SCL-90), and 13.05% using Children Depression Inventory (CDI)^16^. Among children, bullies, victims, and bully-victims are at risk for engaging in depression, anxiety, low self-esteem and delinquency^17^. Physical fighting and bullying victimization prevalence is common among in-school adolescents. In this study, the prevalence of physical fight in the last year and bullying in the last month was reported as 31.2% and 31.5% respectively^18^. In supporting the co-occurring nature of physical fight, bullying and victimization, a recently study among adolescents showed the bullies and bully-victims have high risk for cigarette smoking, alcohol consumption and cannabis use^19^. 79.6% of students in some way- from mild to severe- are involved in bullying and about 81% are bullied as victims^20^. Among 15-18-year-old Iranian adolescents, 33% of them had taken part in a physical fight. In overall, our findings are supported by another study^21^.

This study indicated that active smoking was uncommon behavior with a prevalence of 5.9%. A meta-analysis reported the prevalence of cigarette smoking among Iranian adolescents as 2.5-17% prevalence of cigarette smoking among Iranian adolescents has been reported between 2.5 to 17.0% ^22^. This broad range may be related to the variety of the definition of “being a smoker”, the age difference between the study samples and the location of studies.

The prevalence of risk-taking behaviors among students in the other countries is various. For example the result of national Youth Risk Behavior Surveillance (YRBS) showed that in 2011, the last year prevalence of having physical fight was 32.8% among students. Also this report stated that 20.1% of the students had ever been bullied on school property. For example results from the 2011 national Youth Risk Behavior Surveillance (YRBS) indicated that during the 12 months before the survey, 32.8% of students had been in a physical fight, 20.1% had ever been bullied on school property^23^. In South African school students 36.3% of participants were involved in bullying behavior^24^. Overall, 35% of students reported feeling depressed/stressed ≥ 10 d in the past month^25^. Different rates of risk-taking behaviors may be attributed to cultural values of Iranian families as well as probably due to different definitions of time period of surveys.

Considering co-occurrence is an effective approach to prevent high-risk behaviors. A great deal of research has focused on the co-occurrence of risky behaviors,^27^. Involvement in one risky behavior is related to engagement in other risky behaviors^28^. We examined risk-taking behaviors differently and identified five latent classes for all participants. The five latent classes are as follows: (a) low risk, (b) passive smoker, (c violence and aggression taker with passive smoking, (d) violence and aggression taker without passive smoking and (e) high risk. The probability of all variables is high in high-risk classes. These risk-taking behaviors may occur simultaneously. To the best of our knowledge, there are only few studies that have employed the LCA method to detect the latent classes of risk-taking behaviors among in school students. Moreover, researchers have used various variables to subgrouping of students. Some of discussed below.

LCA was employed to subgrouping 3114 students of middle school based on bullying involvement. The authors were able to detect four sub-classes for risk-taking behaviors as follows: 1) victims (15%), bullies (13%), bully-victims (13%) and noninvolved (59%)^29^. In a study among German students, the authors found three distinct latent classes for multiple substance use pattern in adolescents. These classes were: non-users (61.9%), experimenters (29.0%) and multi-user (9.1%). The results of this study showed that experimental use was predicted by bullying (OR=1.69) and other covariates^30^.

The strengths of the present work were its large sample size and high response rate, both of which increase the generalizability of the findings. The present work also had the following limitations: first, due to the Cross-sectional design of the study, causality could not be assessed. Second, the study relied on self-report data, and underreporting of some risk-taking behaviors was expected, even assuring the participants of the anonymity of the questionnaires

## Conclusion

A considerable percentage of students is in the high-risk class, which stresses the necessity of implementing preventive interventions for this stratum of adolescents. Longitudinal studies are required to determine and monitor the incidence rate and pattern of these behaviors and their correlates. The findings of this study can be used for planning and evaluating interventions by considering the risk factors as well as protective factors of risk-taking behaviors.

## Acknowledgments

This nationwide survey was conducted as a national surveillance program. The authors forward their sincere thanks to the large team working with this project in different provinces.

## Conflict of interest statement

All authors declare that they have no conflicts of interest

## Funding

The study was funded by Isfahan University of Medical Sciences. The faculty members of Isfahan University of Medical Sciences were also involved in the design of the study.

## Highlights

- More than half of the students have been engaged in some grade of risk-taking behaviors.
- Age (OR = 1.41), being male (OR = 5.21, depression (OR = 4.58, anxiety (OR=3.36) and screen time (OR = 3.11) associate with high risk class.
- Notable percent of the individuals are violence and aggression takers who need to be addressed by implementing preventive interventions and mental support provision.
- Planning of preventive interventions should take by considering co-occurrence nature of risk-taking behaviors.
